# Survey and Visual Detection of *Zaire ebolavirus* in Clinical Samples Targeting the Nucleoprotein Gene in Sierra Leone

**DOI:** 10.3389/fmicb.2015.01332

**Published:** 2015-12-01

**Authors:** Huan Li, Xuesong Wang, Wei Liu, Xiao Wei, Weishi Lin, Erna Li, Puyuan Li, Derong Dong, Lifei Cui, Xuan Hu, Boxing Li, Yanyan Ma, Xiangna Zhao, Chao Liu, Jing Yuan

**Affiliations:** Institute of Disease Control and Prevention, Academy of Military Medical SciencesBeijing, China

**Keywords:** *Zaire* EBOV, RT-LAMP, sensitivity, specificity, rapid detection, prevalence

## Abstract

Ebola virus (EBOV) can lead to severe hemorrhagic fever with a high risk of death in humans and other primates. To guide treatment and prevent spread of the viral infection, a rapid and sensitive detection method is required for clinical samples. Here, we described and evaluated a reverse transcription loop-mediated isothermal amplification (RT-LAMP) method to detect *Zaire ebolavirus* using the nucleoprotein gene (*NP*) as a target sequence. Two different techniques were used, a calcein/Mn^2+^ complex chromogenic method and real-time turbidity monitoring. The RT-LAMP assay detected the *NP* target sequence with a limit of 4.56 copies/μL within 45 min under 61°C, a similar even or increase in sensitivity than that of real-time reverse transcription-polymerase chain reaction (RT-PCR). Additionally, all pseudoviral particles or non- *Zaire* EBOV genomes were negative for LAMP detection, indicating that the assay was highly specific for EBOV. To appraise the availability of the RT-LAMP method for use in clinical diagnosis of EBOV, of 417 blood or swab samples collected from patients with clinically suspected infections in Sierra Leone, 307 were identified for RT-LAMP-based surveillance of EBOV. Therefore, the highly specific and sensitive RT-LAMP method allows the rapid detection of EBOV, and is a suitable tool for clinical screening, diagnosis, and primary quarantine purposes.

## Introduction

The 2014 Ebola virus (EBOV) outbreak was the largest to date in West African countries (Frieden et al., 2014; Hampton, 2014). It spread through direct contact with infected individuals, or by touching the blood, organs, bodily secretions and fluids, or the contaminated clothes of such people (MacNeil and Rollin, 2012). Because the initial symptoms of ebolavirus infection can be confused with those of other febrile illnesses such as endemic malaria (Chertow et al., 2014), and because the infection cannot be detected rapidly in patients living in remote areas (World Health Organization [WHO], 2014)^1^, the numbers of infected people and deaths were the highest yet recorded. To control infection and to prevent further transmission during outbreaks of filoviruses such as EBOV, rapid detection is therefore essential (Grolla et al., 2005).

Current methods for the detection and diagnosis of EBOV infection include virus isolation, electron microscopy, immunohistochemistry (Zaki et al., 1999), enzyme-linked immunosorbent assay testing (Niikura et al., 2001), reverse transcription-polymerase chain reaction (RT-PCR), serologic testing for IgM/IgG virus-specific antibodies (Ksiazek et al., 1999a; Saijo et al., 2001), and point-of-care biosensors (Baca et al., 2015). In general, when the EBOV viral load in the blood gets to a higher case fatality rate, the detection of antigens as a suitable method is used for laboratory diagnosis (Fisher-Hoch et al., 1992; Ksiazek et al., 1999b). Thus, the World Health Organization recommends real-time RT-PCR as the first choice for EBOV diagnosis. However, inhibitors present in crude biological samples can inactivate the *Taq* DNA polymerase used in PCR-based techniques (de Franchis et al., 1988). Moreover, these methods are relatively complex and require specialized instruments. Thus, to complement PCR-based methods, another rapid, simple, and effective assay is needed.

Loop-mediated isothermal amplification (LAMP) is a one-step nucleic acid detection method developed by Notomi et al. (2000) which relies on autocycling strand displacement DNA synthesis. This novel method is highly specific and sensitive, takes advantage of four or six specific primers to recognize six or eight different sequences of the target gene, and is performed under isothermal conditions in less than 1 h using *Bst* DNA polymerase. Furthermore, LAMP is less influenced by inhibitors present in complex samples than standard PCR, which is highly beneficial for clinical specimens such as blood components, sputum, feces, or body fluids (Kaneko et al., 2007). LAMP assays have been widely applied to genetic diagnoses, the detection of epidemic bacteria (Hara-Kudo et al., 2005; Song et al., 2005) and viruses (Okafuji et al., 2005), fetal sex identification (Hirayama et al., 2013), and parasite recognition (Chen et al., 2011; Kong et al., 2012).

Kurosaki et al. (2007) developed a simple reverse transcription loop-mediated isothermal amplification (RT-LAMP) assay for the detection of *Zaire ebolavirus*, targeting the trailer region of the viral genome. However, this method has yet to be tested in clinical samples. The EBOV genome is approximately 19 kb, and encodes the following seven genes, which are flanked by untranslated regions: nucleoprotein (*NP*), viral structural protein (VSP)35, *VSP40*, glycoprotein, *VP30*, *VP24*, and RNA-dependent RNA polymerase (Ali and Islam, 2015).

*NP* is highly conserved among all EBOV species currently known, and plays an important role in intracellular events such as replication and transcription of the viral genome, and nucleocapsid formation (Ali and Islam, 2015). It is therefore recommended by the World Health Organization for use as a target gene for the RT-PCR assay.

In the present work, we developed a point-of-care RT-LAMP assay targeted to *NP.* Five sets of primers for the detection of EBOV were designed and used in optimization of the RT-LAMP assay. We also evaluated the specificity and sensitivity of the LAMP method. Finally, 417 blood samples collected from patients with clinically suspected infections were analyzed by RT-LAMP and RT-PCR in clinical diagnosis.

## Materials and Methods

### Viruses, RNA Extraction, and Preparation of Templates

Twenty-six genomes of respiratory pathogens including artificial RNAs of Sudan EBOV (Subtype Sudan, strain Gulu), Zaire EBOV and MARV, SARS coronavirus, influenza A H7N9, H1N1, H2N3, human parainfluenza viruses (PIV) type 1/2/3 and 4, adenoviruses (ADV; serotype 3, serotype 5, and serotype 55), respiratory syncytial virus infection RSVA/RSVB, MERS RNA, human metapneumovirus HMPV, human coronavirus HCoV-229E/ HCoV-OC43/HCoV-NL63, and HCoV-HKU1, bocavirus BoV, as well as three respiratory bacterial pathogens such as *Legionella pneumophila* 9135, *Mycobacterium tuberculosis* 005, and *Haemophilus influenza* ATCC 49247 were used in this study. Total viral RNAs were extracted from 200 μl of each culture using a QIAamp viral RNA mini kit (Qiagen, Hilden, Germany). All infectious materials were handled in biosafety level 3 facilities.

### Preparation of Artificial EBOV RNA

Preparation of artificial EBOV RNA was performed as described previously (Watanabe et al., 2004) with modifications. Briefly, 663 kb *NP* fragments were synthesized (Sangon Biotech Co., Ltd., Shanghai, China) and cloned into vector pGEM-3Zf(+) with inverse orientation of the T7 promoter sequence (Promega). *In vitro* transcription of artificial EBOV RNA from *NP* subclones was carried out using 50 U of T7 RNA polymerase (Promega) in a 50-μl reaction volume according to the manufacturer’s instructions. The RNA concentration was determined by measuring the optical density at 260 nm (OD260), and the RNA purity was determined by calculating the OD260/OD280 absorption ratio (ratios were ensured to be >1.8). RNA was then dissolved in 20 μL DEPC-treated water, and stored at -70°C before use.

### Primer Design

Based on the *NP* sequences of strain Mayinga deposited in GenBank (accession no. AF086833), we selected potential target regions and further analyzed the sequence using Primer Explorer V4 software^2^ by aligning it with other species of EBOV. We designed specific primer sets for the detection of EBO V in RT-LAMP, with each set including an outer forward primer (F3), an outer backward primer (B3), a forward inner primer (FIP), and a backward inner primer (BIP) linked by a four thymidine spacer (TTTT), which can recognize both sense and anti-sense strands. To accelerate the RT-LAMP reaction, an additional loop primer (LB) was designed. All primers were synthesized commercially (Sangon Biotech Co., Ltd.).

### RT-LAMP Assays

Reverse transcription loop-mediated isothermal amplification reactions were performed using a Loopamp RNA amplification kit (Eiken Chemical Co., Ltd., Tokyo, Japan) in a volume of 25 μL according to the manufacturer’s protocol. Each reaction included 80 pmol of FIP and BIP, 40 pmol of LB, 10 pmol of F3 and B3, and 2 μL template RNA. The reaction was carried out at 61°C for 60–80 min in dry bath incubators.

Reverse transcription loop-mediated isothermal amplification amplified products were detected by turbidity monitoring as well as visual observation. To assess turbidity, the amount of white magnesium phosphate precipitate produced during the LAMP reaction process was monitored using a Loopamp Real-time Turbidimeter (LA-230; Eiken Chemical Co., Ltd., Tochigi, Japan) recording the reaction curves at 650 nm every 6 s with magnesium ion (Mg^2+^) in the reaction buffer (Mori et al., 2001). For visual inspection, tubes containing 1 μl of fluorescent calcein were observed by the naked eye and photographed under natural light or UV light at 365 nm. The color changed from orange to green for a positive reaction, while the negative control remained orange.

### Real-time RT-PCR Assays

To illustrate RT-LAMP detection sensitivity, we targeted a region of *NP* using the Liferiver^TM^ EBOV Real Time RT-PCR Kit (Shanghai ZJ BioTech Co., Ltd.) recommended by the World Health Organization. Thermocycler conditions followed the manufacturer’s instructions. During the amplification process, the fluorescence intensity of the reporter dye (FAM) and a quencher dye (TAMRA) was recorded to calculate the normalized reporter signal, which is linked to the amount of product amplified. The standard curve was drawn using the *in vitro* transcribed RNA standard to calculate the number of RNA copies in viral RNA extracts. The threshold cycle (*C*_t_ value) refers to the number of amplification cycles for the fluorescence to reach the threshold.

### Clinical Specimens

A total of 417 clinical specimens from whole blood or swabs were collected from patients thought to be infected by EBOV during the outbreak in Sierra Leone, from 2014 to 2015. RT-LAMP assays and real-time RT-PCR were performed simultaneously by the China Mobile Laboratory Testing Team in Freetown, Sierra Leone. Information about the clinical samples is listed in **Supplemental Tables S1** and **S2**. This study was carried out in accordance with the recommendations of the Institute of Disease Control and Prevention, China with written informed consent from all subjects. All subjects gave written informed consent in accordance with the Declaration of Helsinki.

## Results

### Optimizing the RT-LAMP Assay

**FIGURE 1:**
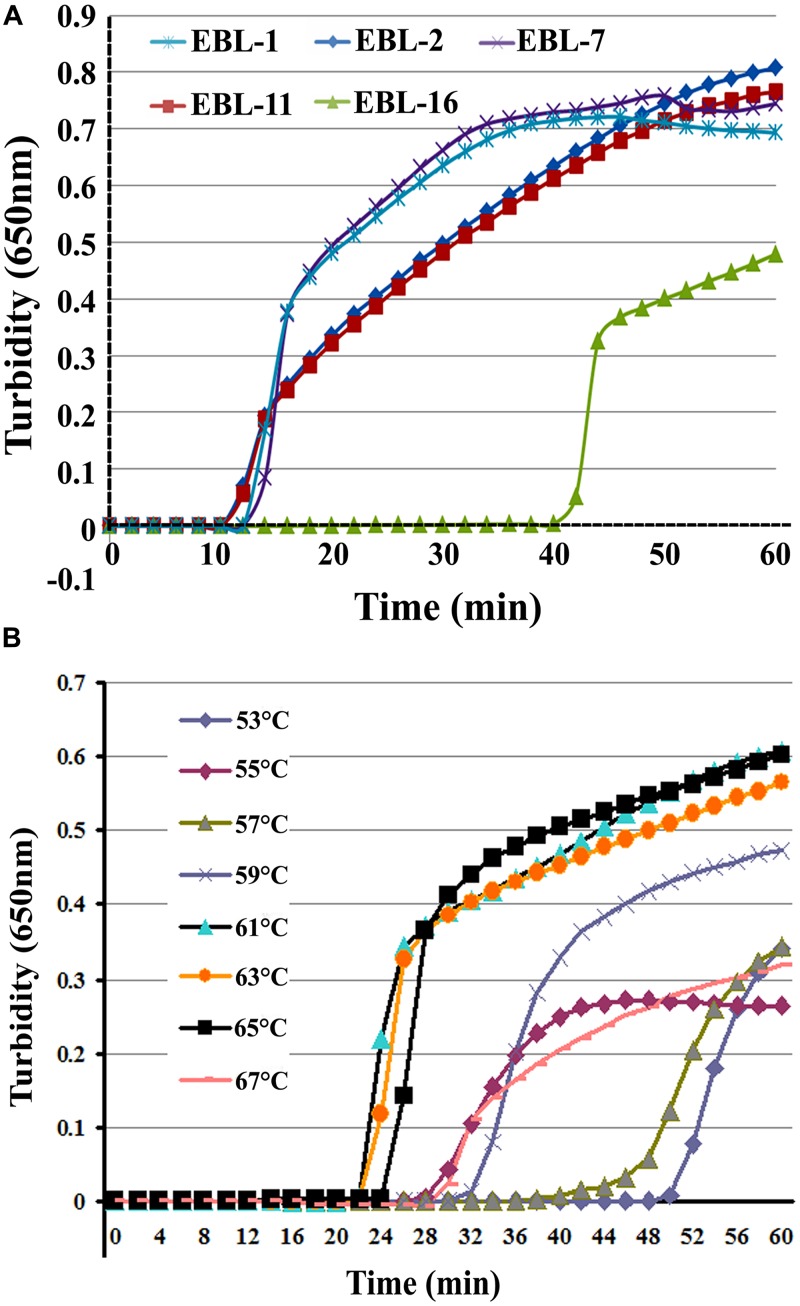
**The most appropriate primers and reaction temperatures for the reverse transcription loop-mediated isothermal amplification (RT-LAMP) assay.** Turbidity was monitored and recorded every 6 s for five sets of primers used to amplify the target gene with a Loopamp real-time turbidimeter at 650 nm. **(A)** A total of five sets of primers including EBL-1, EBL-2, EBL-7, EBL-11, and EBL-16 were designed to detect artificial EBOV RNA. **(B)** Reaction temperatures ranged from 53 to 67°C with 2°C intervals.

A total of five sets of primers were initially designed to detect artificial EBOV RNA using the Real-time Turbidimeter. As shown in **Figure 1A**, the EBL-2 primer set amplified *NP* in the shortest time (∼10 min), so this was chosen as the optimal primer set for RT-LAMP detection of EBOV (**Table 1**).

**Table 1:** Sequence of primers used for specific amplification of *NP*.

Primer	Type	Sequence(5′–3′)
EBL-2F3	Forward outer	GATGGAAGCTACGGCGAAT
EBL-2B3	Backward outer	GTGAGGGCCTGGGACATT
EBL-2FIP	Forward inner	AGTGTCCTCGTCGTCCTCGTCTTAGAGTTACTCGGAAAACGGC
EBL-2BIP	Backward inner	ATCGACCAAGGGTGGACAACAGTTTGTGTCTGTCTGCCCTCTA
EBL-2LB	Loop backward	AAGAACAGTCAAAAGGGCCAGC
EBL-2LF	Loop forward	CCAAGTCATCTGGTGCATTCAT


To further optimize the amplification, we compared reaction temperatures ranging from 53 to 67°C with 2°C intervals. The most suitable reaction temperature range was shown to be 59–65°C (**Figure 1B**), and 61°C was ultimately chosen as the optimal reaction temperature.

### Specificity of *NP* Detection by RT-LAMP

**FIGURE 2:**
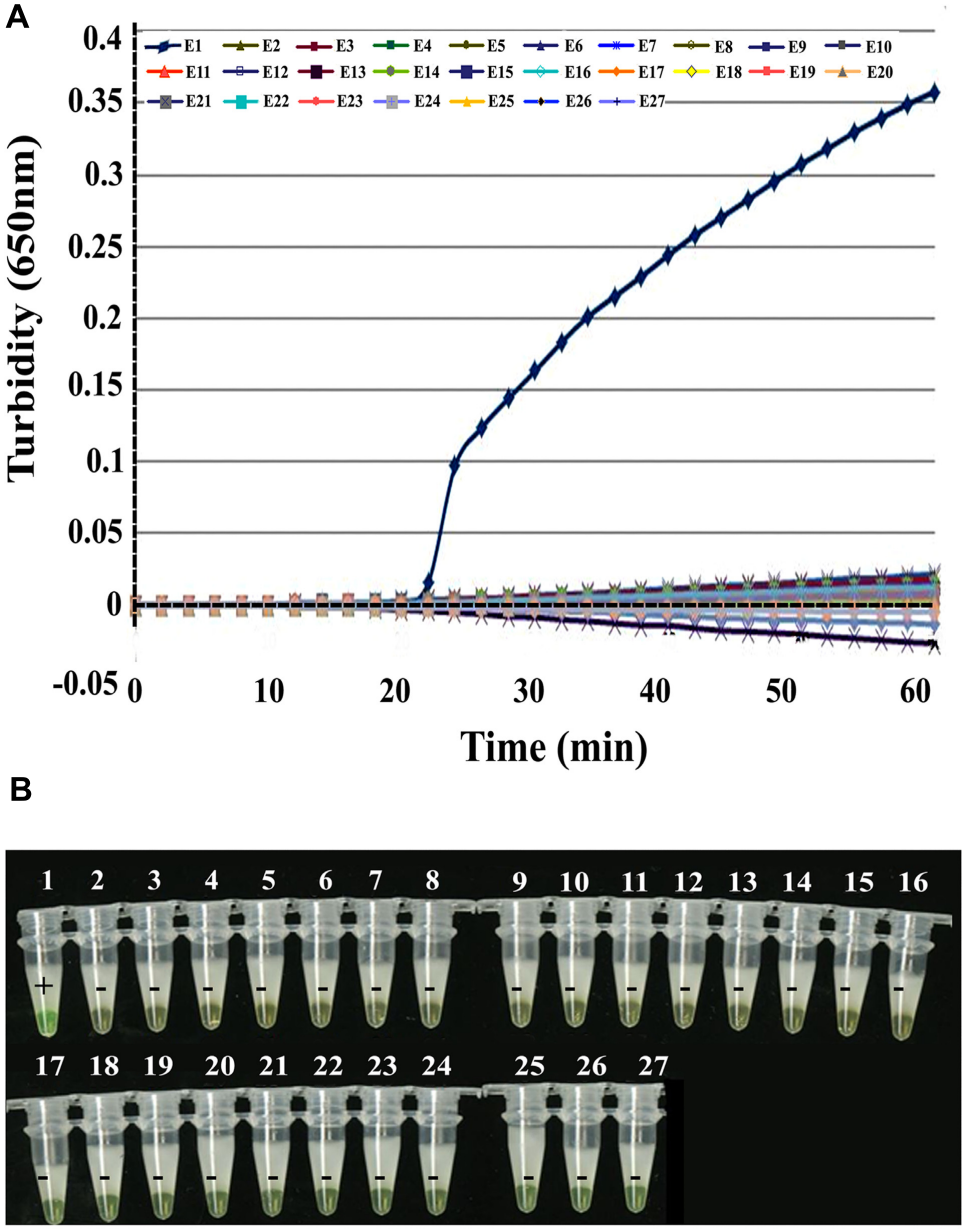
**Specificity of EBOV *NP* detection by RT-LAMP.** **(A)** Turbidity was monitored and recorded every 6 s by a Loopamp real-time turbidimeter at 650 nm. **(B)** Visual detection using a calcein fluorescent detection reagent. Lane 1, positive control (artificial EBOV RNA); lane 2, negative control (double-distilled water); lane 3, Sudan EBOV (artificial Sudan EBOV RNA); lane 4, MARV (artificial MARV RNA); lane 5, SARS coronavirus; lane 6, H7N9; lane 7, H1N1; lane 8, H2N3; lanes 9–12, human parainfluenza viruses (PIV) 1, 2, 3, and 4; lanes 13–15, adenoviruses (ADV; serotypes 3, 5, and 55); lanes 16 and 17, respiratory syncytial virus infection, RSVA, RSVB; lane 18, MERS RNA; lane 19, human metapneumovirus, HMPV; lane 20, bocavirus, BoV; lanes 21–24, human coronavirus, HCoV-229E, HCoV-OC43, HCoV-NL63, and HCoV-HKU1; lane 25, *Legionella pneumophila* 9135; lane 26, *Mycobacterium tuberculosis* 005; and lane 27, *Haemophilus influenza* ATCC 49247.

To test the LAMP specificity for *NP*, we tested 26 non- Zaire EBOV viruses in addition to EBOV itself and *in vitro* transcribed artificial EBOV RNA as the positive control. **Figure 2** shows that EBOV RNA was identified positively by RT-LAMP with the EBL-2 primer set using turbidity monitoring and visual observation. All non- Zaire EBOV strains tested negative, including the blank control, indicating that the RT-LAMP method was specific for EBOV.

### Sensitivity of *NP* Detection by RT-LAMP

**FIGURE 3:**
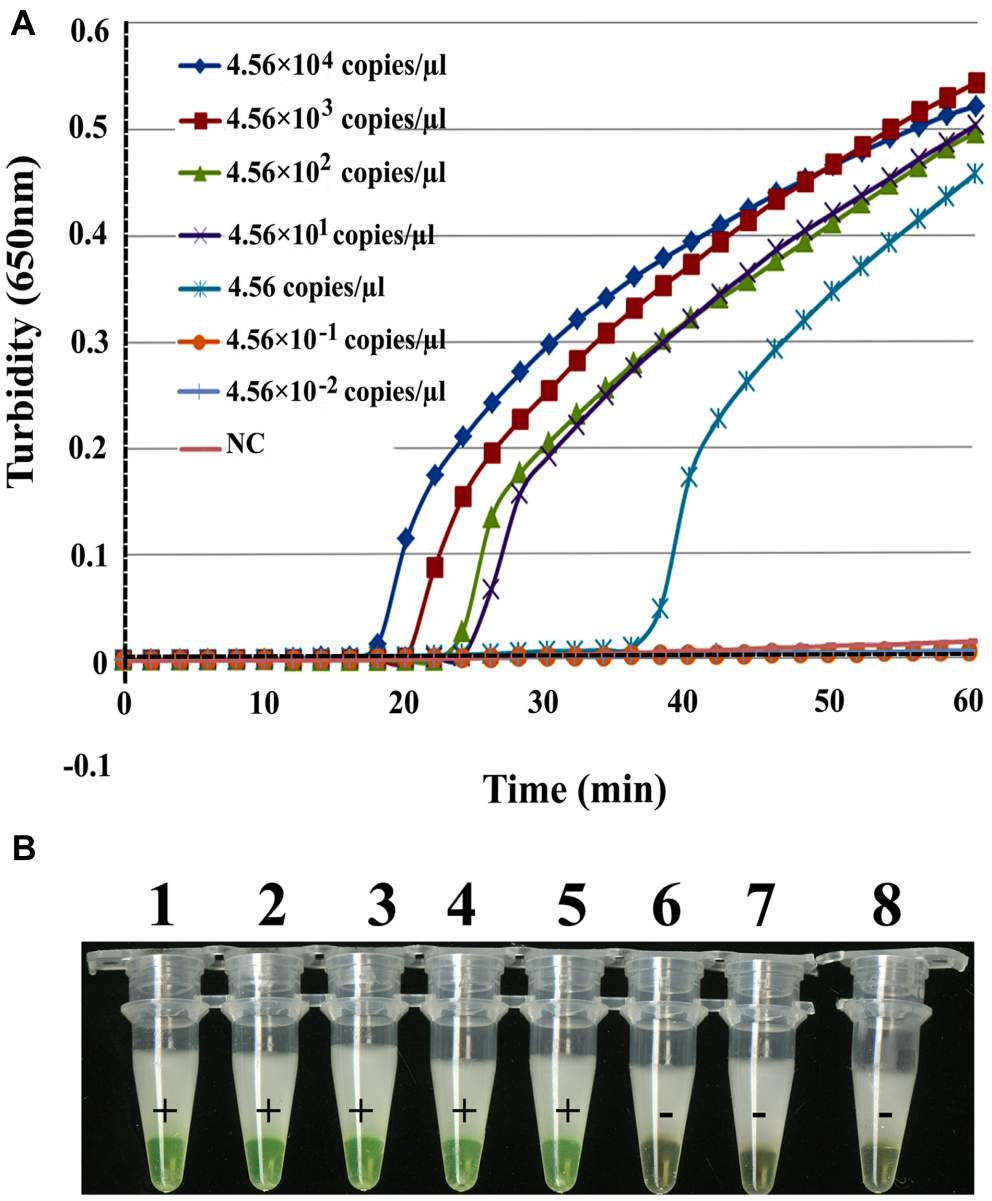
**Comparison of RT-LAMP sensitivities in detecting EBOV *NP*.** Artificial EBOV RNA was serially diluted 10-fold from 4.56 × 10^4^ copies/μL to 4.56 × 10^-2^ copies/μL. **(A)** Turbidity was monitored with a Loopamp Realtime Turbidimeter at 650 nm every 6 s. **(B)** The reaction was detected visually using a calcein fluorescent detection reagent. Artificial EBOV RNA concentrations were: tube 1, 4.56 × 10^4^ copies/μL; tube 2, 4.56 × 10^3^ copies/μL; tube 3, 4.56 × 10^2^ copies/μL; tube 4, 4.56 × 10^1^ copies/μL; tube 5, 4.56 copies/μL; tube 6, 4.56 × 10^-1^ copies/μL; tube 7, 4.56 × 10^-2^ copies/μL; tube 8, ddH_2_O.

To determine the sensitivity of the RT-LAMP assay for EBOV, a series of dilutions were prepared of artificial EBOV RNA ranging from 4.56 × 10^4^ to 4.56 × 10^-2^ copies/μL. As shown in **Figure 3A**, the times of positivity detection ranged from 18 min for 4.56 × 10^4^ copies/μL to 36 min for 4.56 copies/μL of virus RNA by real-time monitoring. Thus, the RT-LAMP detection limit for *NP* is 4.56 copies/μL of artificial RNA in a 61°C reaction lasting for 60 min. For the visual inspection, all positive reactions changed to green while negative ones remained orange under natural or 365 nm UV light (**Figure 3B**). These data indicate that the sensitivity of the two detection methods was the same. The detection limit of real-time RT-PCR for *NP* was 4.56 copies/μL, but this was achieved with a higher *C*_t_ value (*C*_t_ = 41). Thus, we concluded that the sensitivity of the RT-LAMP assay for EBOV was similar or higher than real-time RT-PCR.

### Clinical Sample Detection

**FIGURE 4:**
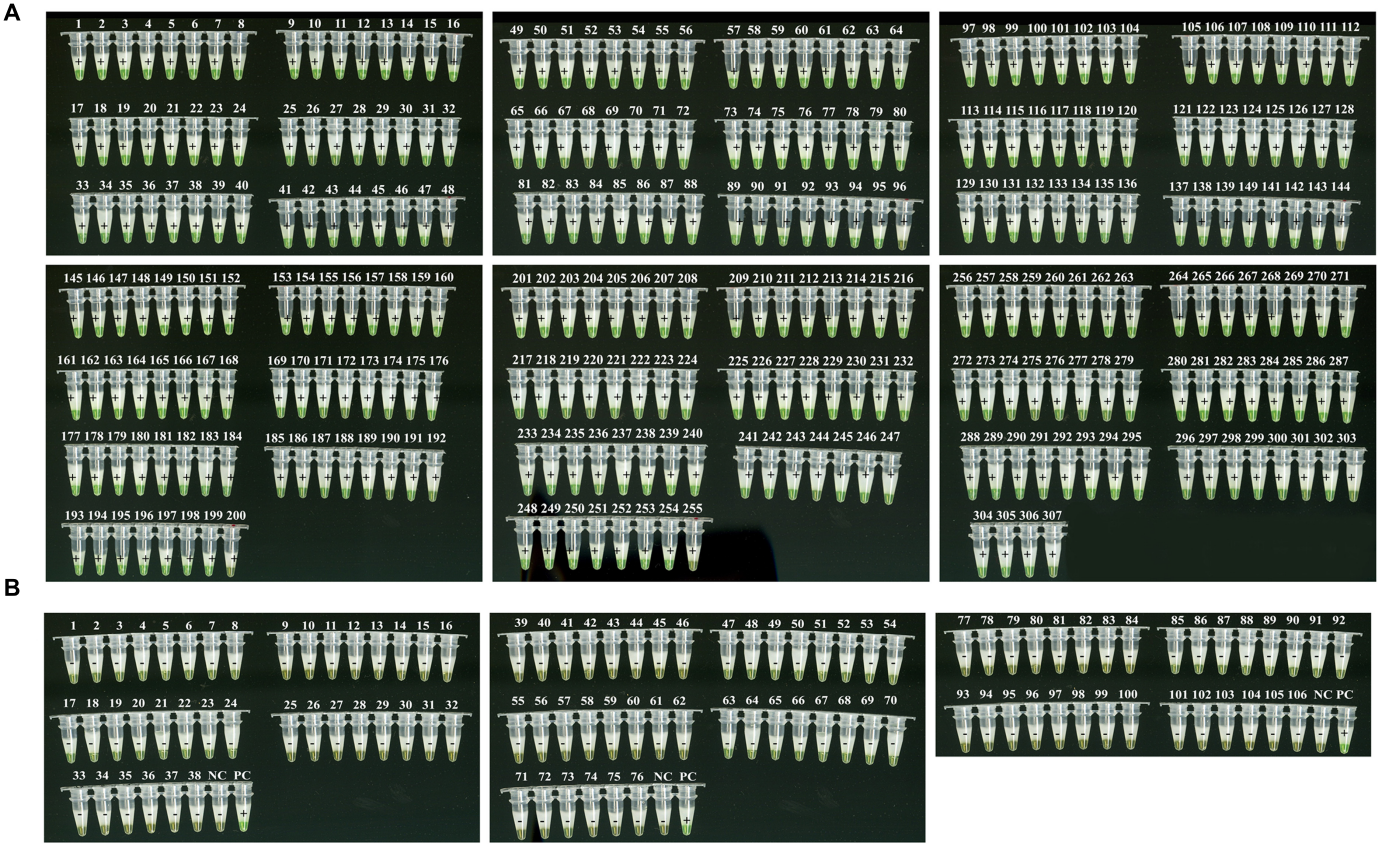
**Visual detection of RT-LAMP findings of 413 patients with clinically suspected infections in Sierra Leone.** Sample information is listed in **Supplemental Tables S1** and **S2**. A total of 307 positive **(A)** and 106 negative **(B)** samples were tested for EBOV infection by real-time RT-PCR and RT-LAMP. NC, negative control (distilled water); PC, positive control (artificial EBOV RNA).

The 417 clinical blood or swab samples were simultaneously analyzed by RT-LAMP and real-time RT-PCR. Of these, 307 patients were confirmed to be infected with EBOV, while 106 tested negative (**Figure 4** and **Table 2**). A higher *C*_t_ value (*C*_t_ > 36) was recorded for the remaining four samples by RT-PCR, and green fluorescence was observed after ∼45 min by RT-LAMP, indicating a low level of EOBV RNA. We suggested that these patients should be monitored for 2 weeks in hospital.

**Table 2:** Reverse transcription loop-mediated isothermal amplification (RT-LAMP) and real-time reverse transcription-polymerase chain reaction (RT-PCR) findings of clinical blood or swab samples.

Results	RT-LAMP	Real-time RT-PCR
Positive samples	307 (Time: 10–30min)	307 (*C*_t_ ≤ 30)
Negative samples	106 (no change in color)	106 (no peak)


## Discussion

Ebola virus has extremely high morbidity and mortality levels in humans, it reemerged and caused an outbreak in Western Africa where 28,476 suspected, probable, and confirmed cases, and 11,298 deaths were reported in Sierra Leone up until Oct. 21 2015 according to Ebola Situation Report from WHO^3^). Although several chemical agents, vaccines, and antibodies inhibit the spread of EBOV in humans and animals, effective therapies for clinical treatment are scarce.

To combat the increasing incidence of EBOV infections, we developed an RT-LAMP assay specific for EBOV diagnosis using primers spanning the 663 bp *NP* sequence of the viral genome. We found that the limit of detection for this technique was 4.56 copies/μL, which compares with 13.4 copies/μL for the Real-Time RT-PCR assay using the Liferiver^TM^-EBOV Kit according to the manufacturer’s instructions. In comparison, the limit of detection was 10 RNA molecule standards targeting the nucleoprotein gene by RT-PCR reported by Weidmann et al. (2004). In the present work, the RT-LAMP assay showed an equivalent or superior sensitivity to RT-PCR assays, indicating that it is sufficiently sensitive to detect low copy numbers of RNA. Furthermore, the RT-LAMP assay showed a high level of specificity, with no cross-reactivity with other species of EBOV or other viruses. It was also comparable with real-time RT-PCR at confirming cases of EBOV infection in clinical samples.

The RT-LAMP assay can detect the presence of virus in a single step in which reverse transcription and DNA amplification proceed in a single tube at a constant temperature of 61°C. Compared with RT-PCR, the sensitivity of the RT-LAMP assay is far greater in the presence of inhibitors. Moreover, RT-LAMP primers specifically recognize target sequences using five independent target sequence regions, compared with RT-PCR primers that recognize only two independent regions. Therefore, the RT-LAMP assay is more suitable for the rapid detection of *NP* in clinical samples.

## Conclusion

We established a rapid and effective visual RT-LAMP assay targeting EBOV *NP*, which we showed to be extremely specific and sensitive in the molecular diagnosis of EBOV infections. It is a reliable tool for the identification of EBOV, so could be used as an alternative method of diagnostic testing at clinical laboratories without the need for special apparatus. Moreover, it can provide accurate results within 1 h, so may be of use in the clinical diagnosis of EBOV in developing countries.

## Author Contributions

JY and XZ conceived and designed the experiments. XW and WL performed clinical detection in Sierra Leone. HL, PL, and DD performed and developed the RT-LAMP. XY, EL, PL, DD, DZ, LC, and XH performed the experiments. XZ wrote the manuscript. JY and XZ edited the manuscript.

## Conflict of Interest Statement

The authors declare that the research was conducted in the absence of any commercial or financial relationships that could be construed as a potential conflict of interest.

## Footnotes

1 http://www.who.int/mediacentre/news/ebola/18-november-2014-diagnostics/en/

2 https://primerexplorer.jp/e/

3 http://apps.who.int/ebola/current-situation/ebola-situation-report-21-october-2015
