# Corrigendum: Survey and Visual Detection of Zaire Ebolavirus in Clinical Samples Targeting the Nucleoprotein Gene in Sierra Leone

**DOI:** 10.3389/fmicb.2016.00948

**Published:** 2016-06-15

**Authors:** Huan Li, Xuesong Wang, Wei Liu, Xiao Wei, Weishi Lin, Erna Li, Puyuan Li, Derong Dong, Lifei Cui, Xuan Hu, Boxing Li, Yanyan Ma, Xiangna Zhao, Chao Liu, Jing Yuan

**Affiliations:** Institute of Disease Control and Prevention, Academy of Military Medical SciencesBeijing, China

**Keywords:** Zaire EBOV, RT-LAMP, rapid detection, sensitivity, specificity, prevalence

Reason for Corrigendum:

In the original article, the patients' names were inadvertently disclosed in the Supplementary Material. The Supplementary Files have since been updated to protect the patients' privacy. The authors apologize for this mistake.

## Author contributions

JY and XZ conceived and designed the experiments. XW and WL performed clinical detection in Sierra Leone. HL, PL, and DD performed and developed the RT-LAMP. YM, EL, PL, DD, DZ, LC, and XH performed the experiments. XZ wrote the manuscript. JY and XZ edited the manuscript.

### Conflict of interest statement

The authors declare that the research was conducted in the absence of any commercial or financial relationships that could be construed as a potential conflict of interest.

