# Supplementary material for: Survey and Visual Detection of Zaire ebolavirus in Clinical Samples Targeting the Nucleoprotein Gene in Sierra Leone
Source: Front Microbiol. 2015 Dec 1;6:1332. doi: 10.3389/fmicb.2015.01332 (PMC4664619; doi:10.3389/fmicb.2015.01332)
Supplement: Supplementary file 1 [file Data_Sheet_1.pdf]

Supplemental Table 1 Information and source of clinical positive samples detected by Real-time RT-PCR and RT-LAMP in Sierra Leone

No. of sample	ID of patients	Source of sample	Date collected	Real-timePCR	RT-LAMP
1	WUR18-280	Blood	2014/11/18	23.21	Yes
2	WUR18-204	Blood	2014/11/18	29.44	Yes
3	WUR-15511	Blood	2014/11/18	35	Yes
4	WUR15576	Blood	2014/11/18	22.54	Yes
5	WU2-1580	Swab	2014/11/18	23.51	Yes
6	WRE-0048	Swab	2014/11/18	32.45	Yes
7	WUR-15514	Swab	2014/11/18	35	Yes
8	WU2-2574	Swab	2014/11/18	34.5	Yes
9		Blood	2014/11/18	20.77	Yes
10	WRE-0050	Swab	2014/11/18	30.14	Yes
11	WUR15527	Blood	2014/11/19	25.63	Yes
12	W/UI-0683	Swab	2014/11/19	28.65	Yes
13	WUI-1600	Swab	2014/11/19	35.5	Yes
14	WRE2059	Swab	2014/11/19	31.1	Yes
15	WRE2570	Swab	2014/11/19	27.02	Yes
16	WUR15528	Whole Blood	2014/11/19	22.47	Yes
17	WU2-3513	Swab	2014/11/19	28	Yes
18	WU2-2079	Swab	2014/11/19	32.3	Yes
19	WUR0830-153	Blood	2014/11/19	32.63	Yes
20	WUR17-149	Blood	2014/11/19	34.8	Yes
21	WUR0831-152	Blood	2014/11/18	30.8	Yes
22	WUR18-285	Blood	2014/11/19	33.5	Yes
23	WUR1478	Blood	2014/11/19	20.28	Yes
24	WUI-0088	Swab	2014/11/19	35.5	Yes
25	WUI-05093	Swab	2014/11/19	29.94	Yes
26	WUR13108	Blood	2014/11/19	21.75	Yes
27	WUR250226	Blood		23.26	Yes
28	WUR15542	Blood	2014/11/21	25.16	Yes
29	WUR15517	Blood	2014/11/21	22.41	Yes
30	WUR15340	Blood	2014/11/21	21.52	Yes
31	WUR250251	Blood	2014/11/21	27.9	Yes
32	WUR250246	Blood	2014/11/21	25.89	Yes
33	WUR250249	Blood	2014/11/21	22.67	Yes
34	WUR15547	Blood	2014/11/21	23.65	Yes
35	WUR250250	Swab	2014/11/21	32.8	Yes

36	WUR250245	Swab	2014/11/21	31.54	Yes
37	WUR13118	Blood	2014/11/21	24.15	Yes
38	W/UI-0685	Swab	2014/11/21	27.83	Yes
39	WUR15544	Blood	2014/11/21	21.68	Yes
40	WUR15545	Blood	2014/11/21	25.07	Yes
41	WUR15543	Blood	2014/11/21	32.14	Yes
42	WUR15514	Blood	2014/11/21	31.12	Yes
43	WUR15539	Blood	2014/11/21	29.99	Yes
44	WUR18-294	Blood	2014/11/22	24.33	Yes
45	WUR18-290	Blood	2014/11/22	31.18	Yes
46	WUR18-293	Blood	2014/11/22	30.97	Yes
47	WUR15550	Blood	2014/11/22	34.2	Yes
48	WUR13122	Swab	2014/11/22	32.39	Yes
49	WRU05186	Blood	2014/11/22	26.59	Yes
50	WRU05187	Blood	2014/11/22	30	Yes
51	158RGH WUR17	Blood	2014/11/22	22.4	Yes
52	139RGH WUR17	Blood	2014/11/22	28	Yes
53	160RGH WUR17	Blood	2014/11/22	34.5	Yes
54	159RGH WUR17	Blood	2014/11/22	27.66	Yes
55	WUR18-305	Blood	2014/11/23	28.88	Yes
56	WUR18-306	Blood	2014/11/23	28.21	Yes
57	WUR18-307	Blood	2014/11/23	28.58	Yes
58	WUR13131	Blood	2014/11/23	22.47	Yes
59	WUR250278	Blood	2014/11/23	21.97	Yes
60	WUR250274	Blood	2014/11/23	26.73	Yes
61	WUI1063	Swab	2014/11/23	23.82	Yes
62	W/UI-05099	Swab	2014/11/23	31	Yes
63	WUR15552	Blood	2014/11/23	33.5	Yes
64	WUR15555	Blood	2014/11/23	29.97	Yes
65	WUR15561	Blood	2014/11/23	33.8	Yes
66	WUR1584	Swab	2014/11/23	30.31	Yes
67	WUR15563	Blood	2014/11/23	22.71	Yes
68	WU2-1582	Swab	2014/11/23	28.59	Yes
69	WUR-15564	Blood	2014/11/23	31.65	Yes
70	WRE0054	Swab	2014/11/23	27.34	Yes

71	WUR155530	Swab	2014/11/23	29.34	Yes
72	WUR15558	Blood	2014/11/23	24.9	Yes
73	WRE2582	Swab	2014/11/23	32	Yes
74	WU2-2088	Swab	2014/11/23	28.03	Yes
75	WRE2581	Swab	2014/11/23	27.9	Yes
76	WUR18-316	Blood	2014/11/24	27.04	Yes
77	WUR18-312	Blood	2014/11/24	29.52	Yes
78	WUR18-331	Blood	2014/11/24	24.8	Yes
79	WUR18-315	Blood	2014/11/24	27.44	Yes
80	WRE2078	swab	2014/11/24	29.31	Yes
81	WUI0101	swab	2014/11/24	26.34	Yes
82	WRE2079	swab	2014/11/24	27.33	Yes
83	WRE2080	swab	2014/11/24	30.41	Yes
84	W/UI-0687	swab	2014/11/24	25.87	Yes
85	W/UI-0690	swab	2014/11/24	30.46	Yes
86	WRE3036	swab	2014/11/24	33.5	Yes
87	W/UI-15028	swab	2014/11/24	26.36	Yes
88	WRU05198	Blood	2014/11/24	23.92	Yes
89	WRU05199	Blood	2014/11/24	23.91	Yes
90	WRU05197	Blood	2014/11/24	26.46	Yes
91	WRU05193	Blood	2014/11/24	33	Yes
92	WRU05189	Blood	2014/11/24	26.49	Yes
93	WRU05192	Blood	2014/11/24	27.08	Yes
94	WRU05196	Blood	2014/11/24	31.07	Yes
95	WUR250280	swab	2014/11/24	28.28	Yes
96	WUR250283	Blood	2014/11/24	28.57	Yes
97	WUR250282	Blood	2014/11/24	23.13	Yes
98	WUR17-162RGH	Blood	2014/11/24	25.92	Yes
99	WUR17-1248 RGH	Blood	2014/11/24	23.04	Yes
100	WUR17-163 RGH	Blood	2014/11/24	34.94	yes
101	WUR17-168 RGH	Blood	2014/11/24	26.12	Yes
102	WUR17-167 RGH	Blood	2014/11/24	30.06	Yes
103	WUR17-170 RGH	Blood	2014/11/24	28.64	Yes
104	WUR15566	swab	2014/11/24	28	Yes

105	WU2-1585	swab	2014/11/25	26.21	Yes
106	WUR15570	swab	2014/11/25	34.16	Yes
107	WUR15569	swab	2014/11/25	26.95	Yes
108	WUR13135	Blood	2014/11/25	18.86	Yes
109	WUR18-346	Blood	2014/11/25	25.58	Yes
110	WUR18-340	Blood	2014/11/25	22.06	Yes
111	WUR18-347	Blood	2014/11/25	25.09	Yes
112	WUR18-343	Blood	2014/11/25	24.73	Yes
113	WUR18-335	Blood	2014/11/25	28.29	Yes
114	WUR17-176	Blood	2014/11/26	22.47	Yes
115	WUR17-174	Blood	2014/11/26	34	Yes
116	W/UI050103	Swab	2014/11/26	30.21	Yes
117	WUR17-179	Blood	2014/11/26	26.4	Yes
118	WRE2081	Swab	2014/11/26	28.45	Yes
119	WRE3037	Swab	2014/11/26	33	Yes
120	WRE3041	Swab	2014/11/26	29.98	Yes
121	WRE3040	Swab	2014/11/26	26.38	Yes
122	WUR18-349	Blood	2014/11/26	26.87	Yes
123	WUR18-353	Blood	2014/11/26	24.06	Yes
124	WUR18-356	Blood	2014/11/27	27.65	Yes
125	WUR18-357	Blood	2014/11/27	23.02	Yes
126	WUR18-358	Blood	2014/11/27	26.74	Yes
127	WUR18-362	Blood	2014/11/27	35.8	Yes
128	WUR18-364	Blood	2014/11/28	35.28	Yes
129	WUR18-373	Blood	2014/11/28	24.6	Yes
130	WUR18-369	Blood	2014/11/28	21.11	Yes
131	WUR18-365	Blood	2014/11/28	36.5	Yes
132	WUR18-371	Blood	2014/11/28	32.77	Yes
133	WUR 250316	Blood	2014/11/27	28.33	Yes
134	WUR 250314	Blood	2014/11/27	20.61	Yes
135	WRE1058	Swab	2014/11/27	25.22	Yes
136	WRE1059	Swab	2014/11/27	27.66	Yes
137	WUR15590	Blood	2014/11/27	20.6	Yes
138	WUR15592	Blood	2014/11/27	27.72	Yes
139	WUR15604	Blood	2014/11/27	26.82	Yes
140	WUR15596	Blood	2014/11/27	20.65	Yes
141	WUR15591	Blood	2014/11/27	33.19	Yes
142	WUR15595	Blood	2014/11/27	23.29	Yes
143	WUR15605	Blood	2014/11/27	28.73	Yes
144	WUR15594	Blood	2014/11/27	34.03	Yes
145	WUR15603	Blood	2014/11/27	28.29	Yes
146	WUR0225	Blood	2014/11/27	26.48	Yes
147	WUR0018	Blood	2014/11/27	34.53	Yes
148	WUR250317	Blood	2014/11/28	23.89	Yes

149	WRE 2084	Swab	2014/11/28	29.01	Yes
150	WUR 15607	Swab	2014/11/28	28.86	Yes
151	WUR 0919	Blood	2014/11/28	25.7	Yes
152	WUR 1491	Blood	2014/11/28	27.98	Yes
153	WUR 1296	Blood	2014/11/28	22.31	Yes
154	WUR15608	Blood	2014/11/28	24.11	Yes
155	WUR 15608	Blood	2014/11/28	27.27	Yes
156	WUR 15609	Blood	2014/11/28	31.72	Yes
157	W/UI-050112	Swab	2014/11/28	26.58	Yes
158	W/UI-15035	Swab	2014/11/28	29.89	Yes
159	WUR17-203	Blood	2014/11/28	29.01	Yes
160	WUR17-207	Blood	2014/11/28	26.28	Yes
161	WUR17-204	Blood	2014/11/28	29.33	Yes
162	WUR17-202	Blood	2014/11/28	27.46	Yes
163	WUR18-379	Blood	2014/11/29	26.65	Yes
164	WUR18-381	Blood	2014/11/29	28.97	Yes
165	WUR18-377	Blood	2014/11/29	26.71	Yes
166	WRU05219	Blood	2014/11/29	27.04	Yes
167	WRU05218	Blood	2014/11/29	22.37	Yes
168	WRU05222	Blood	2014/11/29	27.09	Yes
169	WUR05221	Blood	2014/11/29	23.23	Yes
170	WRU05220	Blood	2014/11/29	20.87	Yes
171	WUR0934	Blood	2014/11/29	33	Yes
172	WUR0937	Blood	2014/11/29	24.97	Yes
173	WUI0116	Swab	2014/11/29	33.73	Yes
174	WRE1060	Swab	2014/11/29	26.47	Yes
175	WRE1061	Swab	2014/11/29	27.53	Yes
176	WU2-25101	Swab	2014/11/29	30.28	Yes
177	WU2 1599	Swab	2014/11/29	27.91	Yes
178	WU2-1598	Swab	2014/11/29	23.95	Yes
179	WUR18-382	Blood	2014/11/29	32.12	Yes
180	WUR18-388	Blood	2014/11/30	19.91	Yes
181	WUR18-380	Blood	2014/11/30	35.5	Yes
182	WUR18-394	Blood	2014/12/1	29.05	Yes
183	WUR18-395	Blood	2014/12/1	21.94	Yes
184	WUR18-392	Blood	2014/12/1	24.67	Yes
185	WUR18-397	Blood	2014/12/1	20.52	Yes
186	WUR18-398	Blood	2014/12/1	20.22	Yes
187	WRE3055	Swab	2014/12/1	28.41	Yes
188	WUR15639	Blood	2014/12/1	21.09	Yes
189	WUR15627	Blood	2014/12/1	26.34	Yes
190	WUR15630	Blood	2014/12/1	22.15	Yes
191	WUR15638	Blood	2014/12/1	24.99	Yes
192	WUR0859	Blood	2014/12/1	23.35	Yes

193	WUR15637	Blood	2014/12/1	29.77	Yes
194	WUR15641	Blood	2014/12/1	30.82	Yes
195	WUR15636	Blood	2014/12/1	26.71	Yes
196	WUR17-224	Blood	2014/12/1	27.95	Yes
197	WUR17-226	Blood	2014/12/1	22.17	Yes
198	WUR17-220	Blood	2014/12/1	28.79	Yes
199	W/UI25051	Swab	2014/12/1	27.98	Yes
200	WUR17-219	Blood	2014/12/1	26.78	Yes
201	WUR17-223	Blood	2014/12/1	25.64	Yes
202	WUR15644	Blood	2014/12/2	31.95	Yes
203	WU2-3008	Swab	2014/12/2	28.05	Yes
204	WU2-3009	Swab	2014/12/2	31.34	Yes
205	W/UI050119	Swab	2014/12/2	26.14	Yes
206	WUI0117	Swab	2014/12/2	32.15	Yes
207	WUR250339	Swab	2014/12/2	31.89	Yes
208	WUR250338	Blood	2014/12/2	28.64	Yes
209	WUR250337	Blood	2014/12/2	22.02	Yes
210	WUR18-399	Blood	2014/12/2	27.91	Yes
211	WUR18-401	Blood	2014/12/2	23.31	Yes
212	WUR13171	Blood	2014/12/2	15.99	Yes
213	WUR13168	Blood	2014/12/2	23.42	Yes
214	WUR13174	Blood	2014/12/2	29.22	Yes
215	WUR15647	Blood	2014/12/2	29.52	Yes
216	WUR15648	Blood	2014/12/2	23.22	Yes
217	WUR15646	Blood	2014/12/2	30.08	Yes
218	WUR13176	Blood	2014/12/2	20.8	Yes
219	WUR13175	Blood	2014/12/2	24.65	Yes
220	WUR250209	Swab	2014/12/2	32.51	Yes
221	WUR250345	Blood	2014/12/2	20.99	Yes
222	WUR250309	Swab	2014/12/2	32.5	Yes
223	WUR250346	Swab	2014/12/2	21.4	Yes
224	W/UI15045	Swab	2014/12/2	28.86	Yes
225	W/UI06705	Swab	2014/12/2	32.02	Yes
226	W/UI25053	Swab	2014/12/2	34.5	Yes
227	WUR17-229	Blood	2014/12/2	32.69	Yes
228	WUR17-237	Blood	2014/12/2	30.35	Yes
229	WUR17-234	Blood	2014/12/2	25.36	Yes
230	WUR17-235	Blood	2014/12/2	26.47	Yes
231	WUR17-230	Blood	2014/12/2	33.2	Yes
232	WUR15630	Swab	2014/12/3	24.33	Yes
233	WUR15655	Blood	2014/12/3	28.73	Yes
234	WUR15650	Blood	2014/12/3	23.32	Yes
235	WUR15652	Blood	2014/12/3	30.86	Yes
236	WUR250355	Swab	2014/12/3	28.9	Yes

237	WUR15740	Blood	2014/12/13	31.55	Yes
238	WUR250350	Blood	2014/12/3	17.8	Yes
239	WUR250349	Blood	2014/12/3	27.62	Yes
240	WUR250351	Blood	2014/12/3	30.15	Yes
241	WUR250354	Blood	2014/12/3	21.1	Yes
242	WUR18-402	Blood	2014/12/3	28.95	Yes
243	WUR18-404	Blood	2014/12/3	20.34	Yes
244	WRU05240	Blood	2014/12/4	19.4	Yes
245	WRU05237	Blood	2014/12/4	21.77	Yes
246	WUR18-409	Blood	2014/12/4	29.45	Yes
247	WUR18-405	Blood	2014/12/4	26.21	Yes
248	WUI0125	Swab	2014/12/4	30.06	Yes
249	W/UI-06708	Swab	2014/12/4	26.7	Yes
250	WUR17-242	Blood	2014/12/4	30.95	Yes
251	WUR25055	Swab	2014/12/4	28.35	Yes
252	WUR15739	Blood	2014/12/13	23.17	Yes
253	WUR17-245	Blood	2014/12/4	21.29	Yes
254	WUR17-243	Blood	2014/12/4	30.21	Yes
255	WUR17-246	Blood	2014/12/4	21.2	Yes
256	WUR18-414	Blood	2014/12/5	22.83	Yes
257	WUR250367	Blood	2014/12/5	22.02	Yes
258	WUR250370	Blood	2014/12/5	25.33	Yes
259	WUR17-248	Blood	2014/12/5	25.31	Yes
260	WUR18-420	Blood	2014/12/6	28.49	Yes
261	WUR17-427	Blood	2014/12/7	18.79	Yes
262	WUR18-420	Blood	2014/12/7	26.91	Yes
263	WUR18-421	Blood	2014/12/7	16.73	Yes
264	WUR18-425	Blood	2014/12/7	29.73	Yes
265	WUR18-423	Blood	2014/12/7	28.39	Yes
266	WUR17-270	Blood	2014/12/7	19.59	Yes
267	WUR17-267	Blood	2014/12/7	20.58	Yes
268	WUR17-271	Blood	2014/12/7	18	Yes
269	WUR17-265	Blood	2014/12/7	31.92	Yes
270	WUR17-264	Blood	2014/12/7	29.37	Yes
271	WUR17-266	Blood	2014/12/7	28.17	Yes
272	WU2-1634	Swab	2014/12/7	20.58	Yes
273	WUI-0131	Swab	2014/12/7	27.43	Yes
274	WUR17-263	Blood	2014/12/7	20.56	Yes
275	WUI1071	Swab	2014/12/7	29.46	Yes
276	W/UI050137	Swab	2014/12/7	33.06	Yes
277	WUR15705	Blood	2014/12/8	32.5	Yes
278	WUR15704	Blood	2014/12/8	22.38	Yes
279	WUR18-431	Blood	2014/12/9	18.6	Yes
280	WUR17-283	Blood	2014/12/9	21.01	Yes

281	WUR17-287	Blood	2014/12/9	21.29	Yes
282	W/UI06717	Swab	2014/12/9	29.24	Yes
283	W/UI15058	Swab	2014/12/9	23.68	Yes
284	W/UI25068	Swab	2014/12/9	21.69	Yes
285	WUR18-442	Blood	2014/12/10	23.19	Yes
286	WUR13196	Swab	2014/12/10	22.81	Yes
287	WRE3066	Swab	2014/12/10	27.25	Yes
288	WUR15722	Swab	2014/12/10	19.23	Yes
289	WUR15721	Swab	2014/12/10	24.24	Yes
290	WUR15723	Swab	2014/12/10	20.19	Yes
291	WUI0143	Swab	2014/12/11	26.14	Yes
292	WUI0145	Swab	2014/12/11	27.64	Yes
293	WRE1576	Swab	2014/12/11	27.58	Yes
294	WUR17-184	Swab	2014/12/11	24.18	Yes
295	WUR10-295	Blood	2014/12/11	24.19	Yes
296	WUR17-293	Blood	2014/12/11	20.17	Yes
297	WUR17-273	Blood	2014/12/11	21.62	Yes
298	WUR17-292	Blood	2014/12/11	19.46	Yes
299	WUR17-294	Swab	2014/12/11	25.36	Yes
300	WUR17-289	Blood	2014/12/11	23.64	Yes
301	WUR17-288	Blood	2014/12/11	18.25	Yes
302	WRU1425	Blood	2014/12/11	22.4	Yes
303	WUR18-447	Blood	2014/12/11	20.96	Yes
304	WUR18-452	Blood	2014/12/11	22.25	Yes
305	WUR18-451	Blood	2014/12/11	33.05	Yes
306	WUR15737	Blood	2014/12/12	29.73	Yes
307	WUR15738	Blood	2014/12/13	19.52	Yes
