# Supplementary material for: Survey and Visual Detection of Zaire ebolavirus in Clinical Samples Targeting the Nucleoprotein Gene in Sierra Leone
Source: Front Microbiol. 2015 Dec 1;6:1332. doi: 10.3389/fmicb.2015.01332 (PMC4664619; doi:10.3389/fmicb.2015.01332)
Supplement: Supplementary file 2 [file Data_Sheet_2.pdf]

Supplemental Table 2 Information and source of clinical negative samples detected by Real-time RT-PCR and RT-LAMP in Sierra Leone

No. of sample	ID of patients	Source of sample	Date collected	Real-time RT-PCR	RT-LAMP
1	WUI-1046	Swab	2014/11/18	No	No
2	WUI-1042	Swab	2014/11/18	No	No
3	WUR-15513	Blood	2014/11/18	No	No
4	WU2-2577	Swab	2014/11/18	No	No
5	WU2-2573	Swab	2014/11/18	No	No
6	W/UI-0679	Swab	2014/11/18	No	No
7	WRE-2056	Swab	2014/11/18	No	No
8	WU2-2575	Swab	2014/11/18	No	No
9	WU2-2576	Swab	2014/11/18	No	No
10	WUR15504	Blood	2014/11/18	No	No
11	WUI-1045	Swab	2014/11/18	No	No
12	W/UI-05089	Swab	2014/11/18	No	No
13	WUI-1051	Swab	2014/11/18	No	No
14	WRE-1538	Swab	2014/11/18	No	No
15	WUR15515	Blood	2014/11/18	No	No
16	WRE-1539	Swab	2014/11/18	No	No
17	WRE-1540	Swab	2014/11/18	No	No
18	WUR250230	Blood	2014/11/18	No	No
19	WUR250227	Blood	2014/11/18	No	No
20	WUR-250226	Blood	2014/11/18	No	No
21	WUR-250229	Blood	2014/11/18	No	No
22	WUR250228	Blood	2014/11/18	No	No
23	WRE-2054	Swab	2014/11/18	No	No
24	WRE-2057	Swab	2014/11/18	No	No
25	WRE-2055	Swab	2014/11/18	No	No
26	WRE-0051	Swab	2014/11/18	No	No
27	WU2-2080	Swab	2014/11/19	No	No
28	W/UI-0684	Swab	2014/11/19	No	No
29	WUI-1601	Swab	2014/11/19	No	No
30	W/UI-0682	Swab	2014/11/19	No	No
31	WUR15524	Blood	2014/11/19	No	No
32	WU2-3040	Swab	2014/11/19	No	No
33	W/UI-0681	Swab	2014/11/19	No	No
34	WUR15526	Blood	2014/11/19	No	No
35	WRE2063	Swab	2014/11/19	No	No
36	WRE2568	Swab	2014/11/19	No	No
37	WRE3028	Swab	2014/11/19	No	No

38	WRE2061	Swab	2014/11/19	No	No
39	WUR17-148	Blood	2014/11/19	No	No
40	WUR17-147	Blood	2014/11/19	No	No
41	WUR17-150	Blood	2014/11/19	No	No
42	WUR17-154	Blood	2014/11/18	No	No
43	WUR18-287	Blood	2014/11/19	No	No
44	WUR18-284	Blood	2014/11/19	No	No
45	WUR18-283	Blood	2014/11/19	No	No
46	WUR18-286	Blood	2014/11/19	No	No
47	WUI-15010	Swab	2014/11/19	No	No
48	WUI-05092	Swab	2014/11/19	No	No
49	WUR-13111	Swab	2014/11/19	No	No
50	WUR13107	Blood	2014/11/19	No	No
51	WUR13110	Swab	2014/11/19	No	No
52	WUR13112	Blood	2014/11/19	No	No
53	WUR13109	Blood	2014/11/19	No	No
54	WUR13113	Blood	2014/11/19	No	No
55	WUR250248	Swab	2014/11/19	No	No
56	WUR250239	Swab	2014/11/18	No	No
57	WUR250244	Blood	2014/11/19	No	No
58	WUR0149	Blood	2014/11/19	No	No
59	WUR18-301	Blood	2014/11/21	No	No
60	WUR18-303	Blood	2014/11/21	No	No
61	WUR18-300	Blood	2014/11/21	No	No
62	WUR18-302	Blood	2014/11/21	No	No
63	WUR15533	Blood	2014/11/21	No	No
64	WUR15515	Blood	2014/11/21	No	No
65	WUR15541	Blood	2014/11/21	No	No
66	WU2-2083	Swab	2014/11/21	No	No
67	WU2-2081	Swab	2014/11/21	No	No
68	WRE0555	Swab	2014/11/21	No	No
69	WUR15513	Blood	2014/11/21	No	No
70	WUR18-295	Blood	2014/11/22	No	No
71	WUR18-298	Blood	2014/11/22	No	No
72	WUR18-289	Blood	2014/11/22	No	No
73	WUR18-291	Blood	2014/11/22	No	No
74	WUR18-297	Blood	2014/11/22	No	No

75	WUR18-299	Blood	2014/11/22	No	No
76	WUR18-296	Blood	2014/11/22	No	No
77	WUR18-288	Blood	2014/11/22	No	No
78	WUR18-292	Blood	2014/11/22	No	No
79	WUR13123	Swab	2014/11/22	No	No
80	WUR15546	Blood	2014/11/22	No	No
81	WUR15551	Blood	2014/11/22	No	No
82	WU2-2087	Swab	2014/11/22	No	No
83	WU2-2086	Swab	2014/11/22	No	No
84	WUR13121	Swab	2014/11/22	No	No
85	WRU05183	Blood	2014/11/22	No	No
86	WRU05184	Blood	2014/11/22	No	No
87	WRU05185	Blood	2014/11/22	No	No
88	WRU05189	Blood	2014/11/22	No	No
89	157RGH WUR17	Blood	2014/11/22	No	No
90	WUR18-304	Blood	2014/11/23	No	No
91	WUR13129	Blood	2014/11/23	No	No
92	WUR13130	Blood	2014/11/23	No	No
93	WUR13128	Blood	2014/11/23	No	No
94	WUR13127	Blood	2014/11/23	No	No
95	WUR13125	Blood	2014/11/23	No	No
96	WUR13126	Blood	2014/11/23	No	No
97	WUR250272-1	Blood	2014/11/23	No	No
98	WUR250272-2	Swab	2014/11/23	No	No
99	WUR250269	Swab	2014/11/23	No	No
100	WUR250273-1	Blood	2014/11/23	No	No
101	WUR250273-2	Swab	2014/11/23	No	No
102	WUR250271-1	Blood	2014/11/23	No	No
103	WUR250271-2	Swab	2014/11/23	No	No
104	WUR250232	Blood	2014/11/23	No	No
105	WUR250276	Blood	2014/11/23	No	No
106	WUR250277	Blood	2014/11/23	No	No
